# Stem Cell-Based Therapies for Multiple Sclerosis: Current Perspectives

**DOI:** 10.3390/biomedicines7020026

**Published:** 2019-03-30

**Authors:** Fernando X. Cuascut, George J. Hutton

**Affiliations:** Baylor College of Medicine, Maxine Mesigner Multiple Sclerosis Center, Houston, TX 77030, USA

**Keywords:** multiple sclerosis, stem cells, autologous hematopoietic, mesenchymal, neuronal, induced pluripotent, human embryonic

## Abstract

Multiple sclerosis (MS) is an inflammatory and neurodegenerative autoimmune disease of the central nervous system (CNS). Disease-modifying therapies (DMT) targeting inflammation have been shown to reduce disease activity in patients with relapsing–remitting MS (RRMS). The current therapeutic challenge is to find an effective treatment to halt disease progression and reverse established neural damage. Stem cell-based therapies have emerged to address this dilemma. Several types of stem cells have been considered for clinical use, such as autologous hematopoietic (aHSC), mesenchymal (MSC), neuronal (NSC), human embryonic (hESC), and induced pluripotent (iPSC) stem cells. There is convincing evidence that immunoablation followed by hematopoietic therapy (aHSCT) has a high efficacy for suppressing inflammatory MS activity and improving neurological disability in patients with RRMS. In addition, MSC therapy may be a safe and tolerable treatment, but its clinical value is still under evaluation. Various studies have shown early promising results with other cellular therapies for CNS repair and decreasing inflammation. In this review, we discuss the current knowledge and limitations of different stem cell-based therapies for the treatment of patients with MS.

## 1. Introduction

Multiple sclerosis is an autoimmune condition characterized by demyelination and the loss of CNS neurons. Active inflammation is most evident in early RRMS. A neurodegenerative process may contribute to disability accumulation during later secondary progressive MS (SPMS) [1]. Available disease-modifying therapies (DMT) for MS have been shown to reduce the number and severity of relapses. Despite the broad range of options, there is still a therapeutic challenge in finding an effective treatment to halt disease progression and to reverse established neural injuries [2,3]. A subgroup of MS patients and those with an aggressive subtype may continue to deteriorate despite the use of DMT [4,5].

In the last 20 years, stem cell transplantation has been considered as a potentially effective therapeutic approach for aggressive MS. At least five types of stem cells have been considered as potential therapeutic approaches: autologous hematopoietic (aHSC), mesenchymal (MSC), neuronal (NSC), induced pluripotent (iPSC), and human embryonic (hESC) [6]. Each of these cell-based therapies works through various mechanisms, including replacing the malfunctioning immune system using immunoablation with high-dose immunosuppressive therapy (HDIT), followed by aHSC administration [7]; modifying the immune response and intrinsic repair mechanisms by alteration of cytokines and trophic factors (MSC) [8,9]; replacing damaged or lost neuronal tissue with NSC or iPSC [10,11]; and providing immunosuppressive and neuroprotective mechanisms (hESC) [12].

In this review, we aim to describe the current knowledge and limitations regarding cell-based approaches for treating MS, and discuss the future considerations that are required to optimize the therapeutic parameters regarding treatment efficacy and safety.

## 2. Immunoablation Followed by Hematopoietic Stem Cell Transplantation

### 2.1. The Procedure

**Figure 1:**
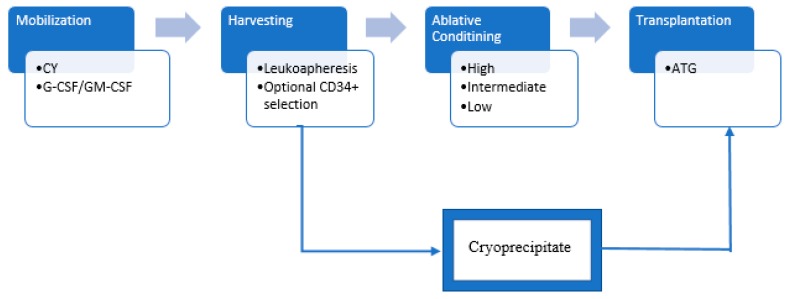
Immunoablation followed by autologous hematopoietic stem cell transplantation. First, HSC are mobilized, typically from peripheral blood, by using granulocyte colony-stimulating factor (G-CSF) or granulocyte-macrophage colony-stimulating factor (GM-CSF), with or without cyclophosphamide (CY). The HSC are then harvested and cryopreserved until the patient is ready for transplantation. The HSC can be positively selected for CD34^+^ cells ex vivo. The next step is to eliminate autoreactive cells (ablative conditioning) using either a high-, intermediate (BEAM), or low-intensity regimen. After the conditioning regimen, the cryopreserved cells are thawed and then infused back into the patient. Anti-thymocyte globulin (ATG) is administered before or after the aHSC infusion to primarily remove any autoreactive T cells that might have escaped prior conditioning procedures. Subsequently, the patient enters an aplastic phase, characterized by an extremely low level of hematopoietic blood cells requiring prophylactic treatment with antivirals and antibiotics.

Immunoablative therapy followed by aHSC therapy (aHSCT) to manage highly active and treatment-refractory MS has been investigated for the last two decades, following reports of the coincidental improvement of autoimmune disease symptoms in patients undergoing transplantation for hematological malignancies [13,14]. The therapeutic effects of immunoablation followed by aHSC transplantation were initially studied in rats with experimental autoimmune encephalomyelitis (EAE)—a rodent model of central nervous system (CNS) inflammation—showing that the procedure can induce remission, prevent relapses, and enhance the recovery of symptoms [15,16,17]. As discussed below, one rationale behind this method is to eliminate the abnormal immune system cells using HDIT, gradually developing a “new” and more tolerant immune system after aHSC infusion [18,19]. An alternative explanation is that HDIT produces a radical and long-lasting suppression of the inflammatory MS activity [20,21,22]. The procedure encompasses a series of steps which includes the mobilization, harvesting, ablative conditioning, and transplantation of aHSC [23,24] (Figure 1). First, hematopoietic stem cell (HSC) are mobilized, typically from peripheral blood, by using either granulocyte colony-stimulating factor (G-CSF) or granulocyte-macrophage colony-stimulating factor (GM-CSF), with or without cyclophosphamide (CY). It is preferred to pretreat with CY due to the potential for either a clinical relapse or worsening of pre-existing symptoms with the administration of G-CSF or GM-CSF alone [25]. The HSC are then harvested and cryopreserved until the patient is ready for transplantation. The HSC can be positively selected for CD34^+^ cells ex vivo, which theoretically reduces the risk of reinfusing potentially autoreactive lymphocytes. However, there is limited evidence indicating any obvious advantage of CD34^+^ selection, which increases the technical complexity of the transplant procedure [26]. The next step is to eliminate autoreactive cells (ablative conditioning) with HDIT. Different conditioning regimens have been proposed and classified according to the intensity of the ablation. High-intensity regimens typically consist of a high IV dose of busulfan, combined with a high dose of CY, and anti-thymocyte globulin (ATG) for in vivo T cell depletion. Total body irradiation is no longer used due to its severe toxicity [26]. Intermediate-intensity regimens involve a combination of different chemotherapies, which often include BCNU, cytosine arabinoside, etoposide, and melphalan, with or without ATG, referred to as BEAM. Other modified BEAM regimens have also been utilized with similar results [27] (Table 1). The low-intensity regimens, also referred to as nonmyeloablative, use CY and ATG [28].

Currently, there is a debate regarding which conditioning regimen should be used. For instance, high-intensity regimens are associated with more adverse effects and mortality [29,30,31,32,33]. Conversely, low-intensity regimens are associated with reduced toxicity and require less supportive care, but may be associated with the early reappearance of MRI lesion activity post-transplant [34,35,36,37]. It is becoming more acceptable to use BEAM or modified BEAM as an intermediate regimen for ablative conditioning [38]. After the conditioning regimen, the cryopreserved cells are thawed and then infused back into the patient. ATG is administered before or after the HSC infusion, to primarily remove any autoreactive T cells that might have escaped prior conditioning procedures. Subsequently, the patient enters an aplastic phase, characterized by an extremely low level of hematopoietic blood cells, requiring prophylactic treatment with antivirals and antibiotics.

### 2.2. Immune Mechanisms

Although the efficacy of aHSCT in MS is increasingly being validated, the immune mechanisms that account for its therapeutic effects are still unclear. There are at least two basic hypotheses to explain its beneficial effects. First, aHSCT is considered to “reset” the immune system by depleting adaptive immune cells and gradually developing a “new” immune system [7,18,19,39,40]. The naïve T cells reemerge over time and undergo a complex T cell receptor (TCR) rearrangement, leading to an augmentation of the T cell repertoire and clonal diversity [18,19,41]. After immune reconstitution, pathogenic CD4+ Th17 lymphocytes are reduced [42]. On the other hand, T cell clones that recognize and react to myelin basic protein (MBP) are initially depleted by the conditioning regimen but reappear over time, although it has not established whether these cells are pathological [41]. The extent to which aHSCT leads to an immune reconfiguration is not completely clear, and demonstrating how the immune system resets to a pre-pathological state is scientifically challenging. However, the characteristics that the immune system acquires following aHCST certainly suggest that it has become alternatively programmed. Conversely, an alternate explanation of the therapeutic benefits of aHSCT might be that the conditioning regimen severely depletes T cell populations for an extended time, thus inducing a state of long-lasting immunosuppression incapable of mounting an autoimmune response of clinical significance [20,21,22]. Both of the aforementioned hypotheses may have diverse effects that could be beneficial to MS treatment and, when applied alone, either hypothesis is ineffective or less effective at explaining the benefits of the treatment than when applied in combination. Therefore, the reconciliation of these hypotheses should be achieved in order to obtain optimal therapeutic results [43].

### 2.3. Current Clinical Knowledge

The initial studies using aHSCT to treat MS were performed over two decades ago [13]. Since then, there has been an increasing number of studies looking into this treatment approach. Typically, the primary endpoint is event-free survival (EFS), which is defined as survival without death, or the patient having no evidence of disease activity (NEDA) (i.e., no disability progression, relapse, and/or new lesions on MRI). Earlier studies were conducted mostly in patients with a high level of disability and progressive disease [13,31,44].

For example, a recent retrospective study evaluated the long-term outcomes of patients with MS treated with aHSCT between January 1995 and December 2006 [45]. In this study, the authors established that most patients had progressive MS (78%), mostly with secondary progressive MS (66%). The median Expanded Disability Status Scale (EDSS) score was 6.5, demonstrating moderately advanced disability, on average. The progression-free survival at 5 years was established for the combined group (46%), RRMS subgroup (73%), and the SPMS subgroup (33%). Also, no statistical difference in the risk of progression between primary progressive MS (PPMS) and SPMS was identified. Furthermore, transplant-related mortality (TRM) occurring in the first 100 days after transplantation was 2.8%. The authors concluded that a younger age, RRMS, and fewer prior disease-modifying treatments were associated with better neurological outcomes.

Recent studies have focused on patients with RRMS with an aggressive disease course, given the evidence that aHSCT is most successful in this population [34,36,46,47,48]. The HALT-MS study, in which only patients with RRMS were enrolled, demonstrated a 5-year event-free survival of 69.2% [49,50]. Similarly, a phase II multicenter single-arm trial of patients with aggressive MS who underwent aHSCT transplantation reported an event-free survival at 3 years of 69.6% [51]. Both groups also demonstrated an improvement of their EDSS by 0.5 points or more at their latest follow-up. An additional large single-center trial using nonmyeloablative aHSCT in patients with RRMS and SPMS showed a 4-year relapse-free survival of 80% and progression-free survival of 87% [52]. The study also showed improved disability scores 2 years after aHSCT, mainly in those with RRMS and mild to moderate disability. This benefit was not seen in patients with severe disability due to progressive MS. These three major studies concluded that either HDIT or a nonmyeloablative regimen followed by aHSCT was effective in inducing long-term sustained remissions and disability improvement.

The safety of aHSCT has dramatically improved through the years. The European Bone Marrow Transplantation (EBMT) Registry analysis of patients treated from 1995 to 2000 showed a TRM of 7.3%, while the TRM from 2001 to2007 was 1.3%, further decreasing to 0.7% during 2008–2016, and most recently down to 0.2% [24,38,45]. This improvement is likely related to the better selection of patients (younger patients with RRMS and exclusion of older patients with advanced disability), increased experience with aHSCT treatment, and decreased use of high-intensity regimens [31]. For example, high-intensity conditioning regimens were associated with life-threatening infections [32,33]. Furthermore, some clinical trials using nonmyeloablative conditioning report no mortality associated with the procedure [52]. A recent meta-analysis found that TRM was close to zero in studies that included patients who were younger, had RRMS rather than SPMS along with a lower baseline EDSS, and whose procedure was performed in more recent years, although there was no significant association of TRM with regimen intensity [53]. The authors pointed out a few limitations that may contribute to their results. Regardless, growing evidence suggests intense conditioning and/or extensive T cell depletion protocols increase morbidity and mortality rate [24].

## 3. Mesenchymal Stem Cells 

### 3.1. Preclinical Animal Studies with Mesenchymal Stem Cells

Besides aHSC, the use of MSC has emerged as a potential powerful cellular therapy for inflammatory and neurodegenerative diseases of the CNS, including MS [54,55]. The MSC are multipotent, nonhematopoietic cells that have both immunomodulatory and regenerative properties [56]. Typically, MSC are obtained from the bone marrow, but other tissues have been used, including adipose tissue, umbilical cord blood, and placenta [57,58]. For example, bone marrow MSC (BM-MSC) have been shown to reduce inflammatory responses, stimulate neuronal stem cell differentiation, and promote the regeneration of damaged areas in the CNS [59,60,61,62,63]. These effects are thought to be possible due to peripheral paracrine signals and/or the homing of MSC to injured tissue [64,65].

Studies on EAE have revealed the potential beneficial effects of MSC. EAE mice exhibit multifocal inflammation, variable demyelination, and axonal damage in the CNS via immunization with myelin proteins or immunogenic myelin peptides [65]. The EAE animal model has been helpful in explaining the immunobiological basis of tissue damage in MS and identifying potential therapeutic approaches. When BM-MSC are infused into EAE mice, there is a reduction in the severity of the clinical symptoms and diminished immune cell infiltration, demyelination, and axonal damage [59,60,61,62,63]. These therapeutic effects are seen when MSC are injected intravenously (IV), intraventricular (IVT), and even intraperitoneally (IP) [61,66,67]. However, the optimal route for MSC injection is yet to be determined. Moreover, the therapeutic effects are observed when BM-MSC are injected at disease onset or peak, but are not seen if they are administered during the chronic stage of disease [60].

The exact mechanisms by which BM-MSC mediate their beneficial outcomes are still not completely understood. One potential explanation includes the release of anti-inflammatory and neurotrophic molecules that modulate the immune inflammatory responses targeting the CNS. For instance, EAE mice that received an IVT infusion of exogenous BM-MSC exhibited a significant reduction in disease activity that was associated with the suppression of reactive T cells [67]. In a different study, human BM-MSC infusion in EAE mice was associated with decreased inflammatory myelin-specific Th1 cells and increased anti-inflammatory Th2 cells [59]. These results suggest an immunosuppressive reaction to BM-MSC infusion. Interestingly, IV injection of conditioned media used in MSC (MSC-CM) culture was associated with the symptomatic improvement of EAE mice, suggesting that the beneficial effects were produced by extracellular immunomodulatory molecules secreted by MSC [68].

An additional possibility that may explain the therapeutic effects of MSC is their migration to inflamed tissues. Several studies have tried to describe the ability of MSC to migrate to and repair injured tissue [61,69]. For example, intravenously administered MSC were detected in CNS in proportion to the degree of inflammation [69]. However, the data are limited, and the factors that attract MSC to areas of inflammation have not yet been elucidated.

In addition to their immunomodulatory capabilities, MS might have the potential of influencing neuronal stem cell differentiation and promoting remyelination and axonal survival. For example, MSC promote the differentiation of oligodendrocytes and/or neurons from neuronal stem cells in vitro, in preference to astroglial fate [70]. Similarly, brain dissection and histopathological studies of EAE mice treated with MSC infusion showed an increased number of oligodendrocytes, remyelination of white matter, and improved axonal integrity in injured tissue [59].

By contrast, some studies have reported deteriorative responses and the exacerbation of EAE symptoms with MSC treatment [71]. These findings implicate a different immunobiological mechanism from the previously described possibilities. Moreover, ectopic tissue formation when MS were administered via IVT in an EAE model [72] and malignant transformation are a theoretical concern, although not yet reported in humans [73].

Finally, it is unclear which tissues are the optimal source of MSC. Adipose tissue (AT) has been cited as a superior source because AT-MSC are relatively easier to harvest, have a higher availability, and better expansion capabilities ex vivo than BM-MSC [74]. Further studies are warranted to determine the best source.

### 3.2. Clinical Trials Using Mesenchymal Stem Cells for the Treatment of Multiple Sclerosis

Based on the results of treating EAE, MSC therapy has advanced to clinical trials. The first publication of a clinical trial using MSC in MS patients was in 2007 by Mohyeddin Bonab and colleagues [75]. Their study assessed the safety and efficacy of autologous BM-MSC transplantation in 10 patients with progressive MS. There were no major adverse events reported with a mean follow-up of 19 months. The preliminary report described varying results of EDSS and MRI progression. The authors pointed out the feasibility of MSC therapy to treat MS patients.

Subsequent studies have investigated the potential of MSC therapy in MS patients. These studies have preliminarily shown beneficial results and the safety of the procedure with no major adverse effects reported [76,77,78]. However, all studies had an open-label design, and varied in the source dose and administration route.

In 2010, a group of experts formed the International Mesenchymal Stem Cells Transplantation Study Group (IMSCTSG) in order to have a consensus protocol on the use of MSC for the treatment of MS [8]. The protocol established a phase I/II clinical trial to evaluate MS patients treated with autologous MSC who have not responded to at least one year of treatment with conventional immunomodulatory therapy. Patients are randomized to receive either IV infusion of autologous BM-MSC or suspension media. At 6 months, these treatments are switched between the two cohorts. The primary endpoints are to analyze the safety of MSC therapy in MS patients and to measure the reduction in the number and volume of new enhancing lesions over a 6-month period.

The IMSCTSG protocol has been adapted for use in multiple clinical trials with varying results. For example, Llufriu and colleagues completed a randomized placebo-controlled phase II trial with 9 RRMS patients who had failed conventional therapy [79]. At 6 months, patients treated with an IV infusion of BM-MSC had no statistical differences in clinical outcomes, brain MRI findings, and optical coherence tomography (OCT) measures. However, there was a trend towards a lower mean cumulative number of gadolinium-enhancing lesions on MRI. The authors concluded that BM-MSC are safe and may reduce inflammatory MRI parameters.

As mentioned previously, MSC may have a theoretical risk of malignant transformation. Therefore, recent studies have focused on investigating the feasibility of using autologous MSC-derived neuronal progenitors (MSC-NP) in lieu of BM-MSC to reduce pluripotency and to minimize the risk of ectopic differentiation after CNS transplantation [80,81]. A phase I open-label clinical trial showed that autologous IT transplantation of MSC-NP is safe and well tolerated. In addition, the authors reported an improved EDSS median in their cohort of 20 patients with progressive MS after treatment. Lastly, positive trends (e.g., muscle strength, bladder function) were more frequently observed in the subset of SPMS patients when compared to PPMS patients [82]. Other studies have shown similar results in the safety and efficacy of MSC-NP transplantation, demonstrating a potential therapeutic alternative [83]. However, further studies are needed to determine if transplantation of MSC-NP has better outcomes when compared to BM-MSC.

As noted above, MSC-CM has been investigated for its therapeutic potential in preclinical animal models. Recently, Danbour and colleagues assessed the safety and feasibility of IT treatment with BM-MSC followed by MSC-CM in patients with MS [84]. The study was conducted as an open-label prospective phase I/IIa clinical trial. No serious adverse effects were reported after the treatment. However, due to the small number and heterogeneity of enrolled patients, it was not possible to obtain statistically significant differences in the efficacy measurements (e.g., EDSS score, clinical, cognitive, ophthalmological, and radiological).

To date, cell therapy with MSC has been, overall, well tolerated and safe. Still, it is not known whether this treatment is capable of reducing inflammatory MS disease activity or promoting the regeneration of damaged areas in the CNS.

## 4. Other Stem Cells under Research

### 4.1. Neuronal Stem Cells (NSC)

In the last decade, there has been particular interest in utilizing NSC as a cellular therapy. Adult NSC are found mostly in two regions of the anterior brain: the subgranular zone (SGZ) within the hippocampal dentate gyrus and the subventricular zone (SVZ) lining the lateral ventricles [85,86]. NSC can give rise to neuronal progenitor cells (NPC) to produce neurons, astrocytes, and oligodendrocytes [87,88]. In EAE, NPC can be activated to migrate to inflammatory demyelinating tissue in the CNS, and differentiate into oligodendrocytes, thus having a potential for therapeutic benefits [10].

One major limitation is the limited number of endogenous NSC available to participate in repair mechanisms [89]. For this reason, therapeutic strategies in animal models are looking into the allogenic infusion of NSC. For example, IVT-transplanted NPC at the peak of EAE migrated exclusively into inflamed white matter and subsequently differentiated into oligodendrocytes; however, their direct implication in myelin repair remains unclear [90]. Additionally, other studies have demonstrated the presence of NPC in some chronically demyelinated lesions [91]. This finding suggests that the absence of NPC in pathological tissue is an unsatisfactory explanation for the clinical symptoms. An alternative proposition to explain this phenomenon is that the prevalent inflammatory setting in EAE produces a non-permissive environment for oligodendrocyte precursor cells (OPC) differentiation and function.

The current clinical trial using NPC comes from MSC-NP, as we previously described.

### 4.2. Human Embryonic Stem Cells (hESC)

hESC have been considered for cell therapy, owing to their capability to differentiate into all the germ layer derivatives. Specifically, the ability of hESC to differentiate into neural lineage has attracted scientific attention regarding the potential treatment of MS [92]. For example, transplanting hESC-derived neural progenitors into EAE mice led to diminished clinical symptoms. Histological studies revealed the presence of transplanted neural progenitors in mice brains, however, remyelination and the production of mature oligodendrocytes were not observed. The clinical improvement was hypothesized to result from immunosuppressive and neuroprotective mechanisms [12]. Further studies are required to better understand the mechanism of action of hESC in treating CNS demyelinating disease.

There have been a few published case reports using hESC to treat MS with limited data suggesting the safety of the therapy [93]. However, well-designed clinical trials are required to show the long-term efficacy and safety of hESC in the treatment of MS.

### 4.3. Induced Pluripotent Stem Cells (iPSC)

Lastly, iPSC are pluripotent cells that have been reprogrammed from somatic cells from skin and other tissues. These cells have the potential to become OPC, hence, they may constitute a suitable cellular therapy modality from an autologous source [94]. The therapeutic potential of iPSC is still under research, but some studies suggest that oligodendrocyte precursor-derived iPSC are capable of ameliorating the clinical and pathological features of EAE [95]. However, the therapeutic effect was found to be mostly due to a neuroprotective effect rather than remyelination. Moreover, the efficient differentiation of iPSC toward selected cell phenotypes could represent a great research challenge for directed disease therapy. Finally, recent studies suggest some caution for clinical development due to the potential for malignant transformation and immune rejection [96,97].

## 5. Conclusions

Immunoablative therapy followed by autologous hematopoietic stem cell transplantation should be considered in aggressive and treatment-refractory MS, since this treatment approach has shown increasing evidence of inducing long-term sustained remission and disability improvement. Further studies are needed to characterize which conditioning regimen should be used. On the other hand, MSC have emerged as a potentially powerful and safe cellular therapy for MS, but the dose, route of administration, and therapeutic effects need further exploration and quantification. Other novel forms of stem cell-based therapies for MS are, at this time, only in the early stages, including those based on hESC, iPSC, and NSC.

## Figures and Tables

**Table 1:** Immunoablation regimens.

Intensity	Regimen
High	High dose of busulfan combined with cyclophosphamide and ATG
Intermediate	BEAM (BCNU, etoposide, cytosine arabinoside, melphalan) +/− ATG
Low (nonmyeloablative)	Cyclophosphamide and ATG
